# Patterns of mental healthcare provision in rural areas: A demonstration study in Australia and Europe

**DOI:** 10.3389/fpsyt.2023.993197

**Published:** 2023-02-06

**Authors:** Jose A. Salinas-Perez, Mencia R. Gutierrez-Colosia, Carlos R. Garcia-Alonso, Mary Anne Furst, Hossein Tabatabaei-Jafari, Jorid Kalseth, David Perkins, Alan Rosen, Daniel Rock, Luis Salvador-Carulla

**Affiliations:** ^1^Department of Quantitative Methods, Universidad Loyola Andalucía, Sevilla, Spain; ^2^Health Research Institute, University of Canberra, Canberra, ACT, Australia; ^3^Department of Psychology, Universidad Loyola Andalucía, Sevilla, Spain; ^4^SINTEF Digital, Health Research, Trondheim, Norway; ^5^Centre for Rural and Remote Mental Health, University of Newcastle, Callaghan, NSW, Australia; ^6^Brain and Mind Centre, University of Sydney, Sydney, NSW, Australia; ^7^Australian Health Services Research Institute, University of Wollongong, Wollongong, NSW, Australia; ^8^WA Primary Health Alliance, Subiaco, WA, Australia; ^9^Discipline of Psychiatry, The University of Western Australia, Perth, WA, Australia; ^10^National Centre for Epidemiology and Population Health (NCEPH), Faculty of Health and Medicine, Australian National University, Canberra, Australia

**Keywords:** mental health service, Integrated Atlas, DESDE-LTC, healthcare ecosystem, health planning, rural healthcare, readiness studies

## Abstract

**Introduction:**

Mental healthcare systems are primarily designed to urban populations. However, the specific characteristics of rural areas require specific strategies, resource allocation, and indicators which fit their local conditions. This planning process requires comparison with other rural areas. This demonstration study aimed to describe and compare specialized rural adult mental health services in Australia, Norway, and Spain; and to demonstrate the readiness of the healthcare ecosystem approach and the DESDE-LTC mapping tool (Description and Evaluation of Services and Directories of Long Term Care) for comparing rural care between countries and across areas.

**Methods:**

The study described and classified the services using the DESDE-LTC. The analyses included context analysis, care availability, placement capacity, balance of care, and diversity of care. Additionally, readiness (Technology Readiness Levels - TRL) and impact analyses (Adoption Impact Ladder - AIL) were also assessed by two independent raters.

**Results:**

The findings demonstrated the usability of the healthcare ecosystem approach and the DESDE-LTC to map and identify differences and similarities in the pattern of care of highly divergent rural areas. Day care had a greater weight in the European pattern of care, while it was replaced by social outpatient care in Australian areas. In contrast, care coordination was more common in Australia, pointing to a more fragmented system that requires navigation services. The share between hospital and community residential care showed no differences between the two regions, but there were differences between catchment areas. The healthcare ecosystem approach showed a TRL 8 (the tool has been demonstrated in a real-world environment and it is ready for release and general use) and an AIL of 5 (the target public agencies provided resources for its completion). Two experts evaluated the readiness of the use of DESDE-LTC in their respective regional studies. All of them were classified using the TRL.

**Discussion:**

In conclusion, this study strongly supports gathering data on the provision of care in rural areas using standardized methods to inform rural service planning. It provides information on context and service availability, capacity and balance of care that may improve, directly or through subsequent analyses, the management and planning of services in rural areas.

## 1. Introduction

Rural healthcare is conditioned by the huge demographic and geographical variability between and within regions. There is a wide variety of biotopes, low population densities, scattered population centers, poor accessibility due to limited transport infrastructures and orography, as well as highly variable cultural characteristics (1). Healthcare is conditioned by the lower population thresholds at which health services must be provided, the scarcity and fragmentation of service availability, workforce shortages, and the turnover of clinical and non-clinical staff (2). Furthermore, droughts, floods, plagues and wildfires impact on the physical, built and human environment of rural areas over long periods of time (3, 4). In these areas the loss or the addition of a single healthcare professional may have a huge impact in the local service availability producing a “roller coaster” effect. A flood impacting on the accessibility to services combined by the retirement of a general practitioner (GP) can completely change the conditions of a local health system in a week, which is exceptional in urban environments. These challenges have a higher impact in care for vulnerable populations such as the elderly, Indigenous peoples, migrants, persons with disabilities, and persons with mental health problems (5–7).

The “Orange Declaration on rural and remote mental health” has put the problems of current models of rural mental health and well-being on the table (8). Service models are usually urban-based, top-down, not based on needs, and fragmented. The poor fit of these approaches in urban settings is amplified in rural settings, leading to greater instability and system fragility. Poorer mental health status in rural areas is linked to scarce, fragmented, inaccessible, lower-quality, and overloaded services (6, 9–11). The specific characteristics, which make rural regions unique, require additional efforts from public health agencies to design an intelligent framework for rural planning distinct from urban health (8). Decision support tools could help planners tailor strategies and resource allocation, as well as set indicators and performance standards adapted to local conditions (12). Key to this process is comparison to other rural areas. Due to the huge disparity of contexts, international comparisons could provide information as useful as within country comparisons. For example, the analysis of service availability in highly remote areas with significant Indigenous populations in Canada, Australia and Finland provided contextual information to better understand suicide rates in these regions, such as isolation, low healthcare accessibility, low availability of specific suicide prevention services, and lack of culturally appropriate health care (7). Similarly, key organizational learning can be drawn from the comparison of the impact and response to flooding in rural areas of Australia and other countries (13).

However, the comparison of service provision raises important methodological issues. Standard units of analysis must be defined to ensure like-for-like comparisons within the same area or with other areas, to avoid the so-called commensurability bias in health system research (14). Moreover, there is manifest ambiguity and vagueness in the naming and description of existing services. Similar services may be named differently whilst services named and grouped in the same cluster may undertake very different activities. This terminological variability (14) may overshadow duplication of services and the real magnitude of diversity in the provision of services across different areas. The lack of clarity in the definition of service delivery also hampers the description of common interventions such as psychotherapy (15) or case management (16). Non-commensurability, terminological variability, and ambiguity constitute major sources of systematic bias in health services research, and the magnitude of this problem has remained largely unnoticed until very recently (17).

Thus, care provision studies need a common methodology for the standard description of services that is not based on their official names available in official service directories, webpages, or similar listings. Some approaches have demonstrated their usability in service comparison. For example, the healthcare ecosystem approach provides a framework for the analysis of mental health systems (18, 19) studying their patterns of care through the internationally validated assessment tools such as the Description and Evaluation of Services and DirectoriEs for Long Term Care (DESDE-LTC). This system has shown its utility in the comparison of urban environments (20), the comparison of a rural area with urban areas (21), or in the comparison of service provision in highly remote areas (7). However, there is no study analyzing the usefulness of tools for international service comparison of care provision in rural areas, that is, areas with a typical population density between 1.5 and 300 inhabitants per km^2^.

This demonstration study aimed to describe the specialized adult mental health service provision in different rural environments in Australia, Norway, and Spain, and to demonstrate the suitability, readiness, and impact of the healthcare ecosystem approach and DESDE-LTC to compare rural care throughout different OECD (Organization for Economic Co-operation and Development) countries.

## 2. Materials and methods

### 2.1. Design and study areas

This demonstration study followed a healthcare ecosystem approach where the detailed evaluation of the service provision was encompassed with the analysis of contextual factors (e.g., population characteristics, healthcare system, service provision, etc.) in defined rural health districts (18). Four rural catchment areas with highly divergent biotopes, health policies and patterns of care were selected in Western Europe (Lleida, Spain and Sør-Trøndelag, Norway) and in Australia (South West, Western Australia, and Central Tablelands, New South Wales). These areas were chosen because their mental health service provision had been described previously using the same standardized tool, so they were comparable, and provided case examples of the healthcare models in North/South Europe and Western/Eastern Australia.

#### 2.1.1. South West (Western Australia, Australia)

The South West region is one of the nine in Western Australia. It has an area of 23,970 km^2^, and a population of about 170,000 people (2016). Bunbury is the main city in the region (71,090 people). It is included in the Country WA Primary Health Network (PHN) and the WA Country Health Service (who operate the state specialist services), the main jurisdictional division of the health system in this region. Out of the seven rural and remote regions in WA, South West is the only region with a population over 150,000 with all other areas in this state considered remote except for metropolitan Perth.

#### 2.1.2. Central Tablelands (New South Wales, Australia)

This region is in central New South Wales and covers approximately 31,347 km^2^ and is home to over 165,000 people (2016). It includes the major town of Orange (38,097 residents) and falls predominantly within Wiradjuri Aboriginal country. It is part of the Western NSW Primary Health Network, the national program commissioning primary treatment services in the area and funded by the federal department of health. This PHN corresponds to two Local Health Districts that manage local specialized care and depend on the State Government (NSW-Health).

#### 2.1.3. Sør-Trøndelag (Trøndelag, Norway)

It comprises the southern territory of the Trøndelag county located in central Norway. It covers 18,856 km^2^ with 25 municipalities and is the catchment area of the St Olavs Hospital Health Trust. Slightly under 300,000 people lived in this area (2012). Trondheim is the main city with 176,348 inhabitants. Central Norway Regional Health Authority, one of four state-owned regional health authorities in Norway, is responsible for the specialized mental health services in Sør-Trøndelag and owns the St Olavs Hospital Health Trust. Additionally, municipalities provide primary care, social and long-term care. Sør-Trøndelag provides a case example of service delivery in rural areas of Northwestern Europe.

#### 2.1.4. Lleida (Catalonia, Spain)

The Lleida Health Region is one of the seven health regions in Catalonia (North-East Spain). It covers 5,426 km^2^ and had a population over 365,000 inhabitants (2012). Lleida is its main city with a population of 139,000 inhabitants. The Department of Health is responsible for planning and funding the health system, and the Catalan Health Service (CatSalut) is responsible for ordering the public healthcare system insuring and managing the assigned funding. CatSalut purchases services from providers through healthcare services management contracts. The analysis of the financing system of MH care in the Catalonia region in comparison with the rest of Spain has been published elsewhere (22). Non-health care is provided from other public agencies, such as social affairs, education, employment, and justice. Lleida provides a case example of service delivery in rural areas of Southwestern Europe.

Although urbanization is a key concept for policymakers and administrations and it is broadly used for territorial planning, there is a lack of international consensus in the definition of remote, rural, urban and macro urban areas. International organizations like the European Union, FAO, UN-Habitat, OECD, and The World Bank have developed consensus definitions although differences across these organizations persist (23). Traditional indicators of urbanicity/rurality include the number of inhabitants, population density and proportion of the primary sector in the local economy. To avoid the distortion introduced by the size of the study areas, which reduced the comparability between countries, the new definitions use a smaller study unit with a fixed size (1 × 1 km^2^). Thus, a rural area could be defined by a cluster of grids with a population density under 300 inhabitants/km^2^ or less than 5,000 inhabitants. The classification of the grids included in the study areas can be interactively consulted using the Global Human Settlement Layer website (24). According to this classification, the South West region of Western Australia and Central Tablelands of New South Wales are mostly composed of rural grids with a few town and suburb grids, while Lleida and Sør-Trøndelag (Western Europe) also have a majority of rural grids, but with a higher number of urban center grids (Lleida and Trondheim). Thus, the four study catchment areas are mainly rural, but their main population center is a town or a city.

Likewise, the distinction between rural and remote areas is highly relevant for health planning. Remoteness has effects on the population mental health status and wellbeing different from rurality (6, 25). However, a worldwide standard definition of remoteness is missing (26). In a previous study on remote service provision we selected areas with an extremely low population density (under 1.5 inhabitants per km^2^) (7). Following this criterion, there are remote areas in northern Norway and in western and inner Australia, but not in Spain. For purposes of comparison, areas with a population density well over this cut-off were selected from the Western Australia and the Western NSW mental health atlases for this study (27).

### 2.2. Standard description of mental health services

We used the DESDE-LTC instrument to conduct the standard description of services in these regions. DESDE-LTC has been used in a range of international service research projects (28) to overcome the commensurability and the terminological bias problems in service research. Its development, validation, and structure has been described elsewhere (29, 30).

In brief, services are disaggregated in one or several “Basic Stable Inputs of Care” (BSIC), which is the minimal organizational unit composed by care teams with temporal and organizational stability arranged for delivering care to a defined population in a catchment area. The BSIC is described by one or more “Main Types of Care” (MTC) according to their most significant activity. MTCs are organized in a tree diagram with six main branches: residential care, day care, outpatient care, accessibility to care, information for care, and self-help and voluntary care. These branches are divided into sub-branches considering different key characteristics (emergency/continuing care, team’s professional level, time intensity, length of stay, mobility among others). The last sub-division of this hierarchical structure gives the final description of the BSIC with its MTC code or codes. A DESDE-LTC code thread is then produced including information about the type of catchment area, the defined target population, CIE-11 diagnoses, the MTC and additional qualifiers that provide information that could help to differentiate BSICs with the same code. A glossary of terms, codes, and descriptions is published elsewhere (7, 31).

The inclusion criteria in this study were services: (1) targeting adults with a lived experience of mental illness; (2) not having a significant out-of-pocket cost; (3) having a temporal stability of at least 3 years; (4) having its own administrative support, space, finances, and documentation; (5) delivering care to the study area; and (6) providing direct care or support to consumers (i.e., financing services were excluded from this analysis).

### 2.3. Readiness and impact analysis

Readiness is the level of preparedness for the application of a new scientific knowledge for commercialization or generalized use in the real world (32). The technology readiness levels (TRLs) are a systematic measurement that supports assessments of the maturity of a particular technology during the early implementation phase of a program or a tool. Nine levels are considered. The levels adapted to public health are: TRL 1, Basic principles observed and reported; TRL 2, Technology concept and/or application formulated; TRL 3, Proof of concept; TRL 4, Prototype completed; TRL 5, Validation of the prototype in relevant environment; TRL 6, Pilot in a relevant environment; TRL 7, Demonstration in a real world environment; TRL 8, Actual system completed and release preparation (pre-release); TRL 9, Actual system “flight proven,” released and/or commercialized. DESDE-LTC tool was evaluated according to this scale by two experts (MAF and LSC) in each study jurisdiction.

Finally, the adoption of the four case studies was assessed with the Adoption Impact Ladder (AIL) (33). This is an inventory for evaluating the level to which the target organization has taken the application of new knowledge as its own. It uses a quasi-ordinal scale with seven categories: (0) no adoption; (1) awareness; (2) assimilation; (3) conversion (or translation); (4) allocation of funding; (5) provision of resources; and (6) routinization (or monitoring). The studies were evaluated by two international experts (CRGA and LSC).

### 2.4. Data collection and statistical analysis

Service data for South West, Central Tablelands and Lleida was collected for the Integrated Atlases of Mental Health developed in each territory in the framework of the GLOCAL project (Global and Local Observation and mapping of CAre Levels) (27). South West region service provision was studied in the Integrated Atlas of Mental Health and Alcohol and Other Drugs of Western Australia in 2016 (34) and the Central Tableland region data was gathered from the Integrated Atlas of Mental Health of Western NSW in 2016 (35). The service directory of Lleida was retrieved from the Integrated Atlas of Catalonia in 2012 (36). The mental health service directory of Sør-Trøndelag was collected as part of the European project REFINEMENT in 2012 (37). All these atlases followed the same data collection methodology and three members of the team participated in all these studies (LSC, JASP, MRGC).

The comprehensive service directory in each area was built through meetings with local and regional public officers, stakeholders from different sectors, the review of public service lists, and a survey conducted to draw service data through face-to-face or phone interviews, and online questionnaires with all managers of the services identified in every catchment area. Besides the service information, demographic and socioeconomic data was collected from the respective National Statistical Agencies to know the contextual factor of the study mental health system.

The study of care provision was conducted first, with the calculation of a range of rates per 100,000 inhabitants to analyze the availability of MTCs and placement capacity by large DESDE-LTC code groups. Second, came the analysis of the balance of care that is the proportion of codes delivering health-related or social-related care. Third, was the analysis of the diversity of care, another indicator that shows the variability of mental healthcare through the count of different codes or MTCs available in the area.

## 3. Results

### 3.1. Context

Table 1 shows the sociodemographic characteristics of the study areas. Study areas have a size between 5.4 and 31.3 thousand square kilometers, and a population density between 5 and 35 inhabitants per square kilometer. The weight of the primary industries sector within the regional economy is relevant in all areas, although it is masked in the European areas because of the existence of a medium size city embedded in the two European areas. Extractive industries (mining) are also remarkable in the two Australian areas. The aging and dependency indexes are similar, although Central Tablelands has the highest figures. Unemployment is lower in Sør-Trøndelag and higher in Lleida, which shows the economic differences between both European countries, while the rates for Australian areas are similar. Finally, it is worth mentioning that Lleida has the highest percentage of foreign residents or Culturally And Linguistically Diverse (CALD) people, mainly linked to the primary sector.

**TABLE 1:** Demographic and Socioeconomic Indicators of the South West and Central Tablelands (Australia), Sør-Trøndelag (Norway) and Lleida (Spain).

	South West	Central Tablelands	Sør-Trøndelag	Lleida
Country/State or region	Australia/Western Australia	Australia/New South Wales	Norway/Trøndelag	Spain/Catalonia
Main city (inhabitants)	Bunbury (71,090)	Orange (38,097)	Trondheim (176,348)	Lleida (139,834)
Total population	171,998	165,233	297,950	367,984
Surface (km^2^)	23,968	31,347	18,856	5,426
Population density	7.18	5.27	15.80	35.80
Aging index (>65/ <15 × 100)	84.03	95.53	81.42	87.22
Dependency index (<15 and >65/15–4 × 100)	59.82	62.96	49.55	49.57
% of unemployment	6.95%	6.23%	2.79%^1^	11.41%
% People working in Agriculture, Forestry and Fishing	6.17%	13.92%	2.95%	11.19%^2^
% People working in mining and quarrying	6.30%	10.79%	1.56%	0.06%
% of non-national citizens	8.03%	3.46%	6.64%	19.35%

Source: demographic data was extracted from statistics agencies. Data from Australian areas refer to 2016, and for the European areas to 2012, except in ^1^2010, and ^2^2011.

### 3.2. Service provision

**FIGURE 1:**
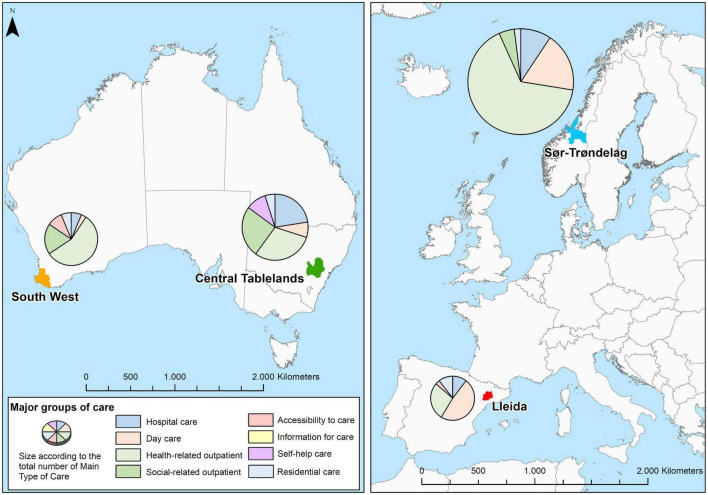
Location of South West and Central Tablelands regions (Australia), Lleida (Spain), and Sør-Trøndelag (Norway), and proportions of mental health care by main branches of the DESDE-LTC system in the four areas.

**FIGURE 2:**
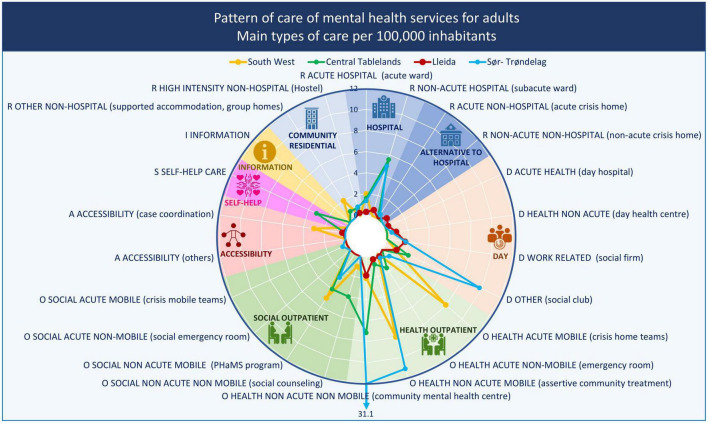
Adult mental health service provision by main type of care (MTC) group in the South West and Central Tablelands regions (Australia), Lleida (Spain), and Sør-Trøndelag (Norway). R, residential care; D, day care; O, outpatient care; S, self-help care. Rates of groups of care for adults per 100,000 adult population.

Table 2 provides a summary of the adult mental health service pattern in each study area in raw numbers and percentages, Figure 1 maps the study areas along with the proportion of major groups of care, and Figure 2 displays the availability rate per 100,000 inhabitants in a greater detail in terms of type of care groups.

**TABLE 2:** Analysis of mental health provision in the study areas.

	South West	Central Tablelands	Sør-Trøndelag	Lleida
Basic Stable Inputs of Care – BSIC (number)	24	39	102	28
Main Types of Care – MTC (number)	32	40	149	29
R – residential care (number and share)	4 12.5%	11 27.5%	17 11.4%	6 20.7%
D – day care (number and share)	1 3.1%	3 7.5%	27 18.1%	14 48.3%
O – outpatient care (number and share)	24 75.0%	22 55.0%	105 70.5%	8 27.6%
A – accessibility to care (number and share)	3 9.4%	0	0	1 3.4%
I – information for care (number and share)	0	0	0	0
S – self-help care (number and share)	0	4 10.0%	Not available	0
Availability of beds by type of facility per 100,000 residents (adults)	Hospital: 26.86 Non-hospital: 24.87	Hospital: 95.26 Non-hospital: 18.26	Hospital: 103.52 Non-hospital: 11.11	Hospital: 18.98 Non-hospital: 26.75
Balance of care (share of health/social care)	63%/38%	53%/48%	77%/23%	59%/41%
Diversity of care (number of different types of care)	10	19	19	15

#### 3.2.1. Residential care

All the study areas had at least one reference hospital with a ward providing acute care. Only Sør-Trøndelag had non-hospital acute care with 24h-physician availability. Subacute care units at a hospital were found in the Norwegian and the Spanish area, and in Central Tablelands. Lleida also had a medium-long stay residential unit. Community residential services were available in all the study areas. However, it was remarkable that low-intensity (or low-supervision) community residential care was only available in the Australian areas. The availability of community residential services rate in Sør-Trøndelag was complemented with generic supported accommodation provided by the municipalities. These generic services were not included in the spider graphs since they were available for any population in need and not exclusively for mental health users. South West also had a non-specialist GP hospital where psychiatric cases could be admitted, but they did not meet the study criteria to be listed as specific mental healthcare services.

The study of the availability showed lower rates of residential care in Lleida, although the rate of beds per inhabitant was similar to South West (Table 2). In contrast, Sør-Trøndelag and Central Tablelands had the highest availability for all DESDE-LTC code groups represented for both MTC and beds. The bed availability rate in subacute hospital units in Central Tablelands was most probably lower since the catchment areas of four units (72 beds) at Bloomfield Hospital were statewide, and an unknown number of admissions came from outside the area. The two Australian areas had higher rates of community residential services of both high and low intensity of support per 100,000 adult inhabitants.

#### 3.2.2. Day care

Day care services varied between the study areas, being more common in Europe than in Australia. Health-related day care was available in Lleida for acute (i.e., day hospitals) and non-acute care and in Sør-Trøndelag for non-acute care, while it was not available in the Australian areas. Likewise, day care codes related to employment were only available in the European areas. Instead, social-related activities were found in every study area. The highest availability rate for non-acute health-related day care belonged to Lleida, for other social-related day care to Sør-Trøndelag, and for work-related care was practically the same in both European areas. Moreover, Central Tablelands had a higher rate of social-related day care than Lleida.

#### 3.2.3. Outpatient care

Outpatient health-related care was the most common type of care, present in all the study areas. However, non-health-related types (social care) were not available in Lleida. On the contrary, mobile outpatient care was available in all the study areas. Regarding availability rates, it is worth mentioning the considerable high values of Sør-Trøndelag for health-related care, especially for non-mobile and non-acute care, and South West for non-acute mobile care. Australian areas also had the greatest availability of non-health (social) outpatient care.

#### 3.2.4. Other care

Finally, the provision of accessibility, information, and self-help care was scarce in all areas. Accessibility care was not available in Sør-Trøndelag and in Central Tablelands. Self-help care was only found in Central Tablelands, although this type of care was not included in the analysis of Sør-Trøndelag. Finally, information for care as a separate service was not identified in any area.

#### 3.2.5. Balance and diversity of care

The balance of care is here measured as the relative weight of the health-related care in comparison with the non-health-related (mainly social care). The rural area with a more balanced care system was Central Tablelands, while in the remaining areas, especially in Sør-Trøndelag, health-related care was dominant over social care.

The diversity of care was measured according to the total number of different DESDE-LTC codes found in each area. Central Tablelands and Sør-Trøndelag showed the highest variability of service types, while South West was the least diverse area, below the area of Lleida that had a lower number of services but more diverse than in South West region of Western Australia.

#### 3.2.6. Studies of readiness and impact assessment

Two experts evaluated the readiness of the use of DESDE-LTC tool in their respective regional studies. All of them were classified in the TRL 8 (The tool has been demonstrated in a real-world environment and it is ready for release, broader use and routinization). Their impact on health organizations was also similar according to the experts. They were scored as level 5 (Resources allocated and tested prior to routinization). The studies in three of the four regions (Australia and Spain) were commissioned by the regional health authorities and had supported the management and planning of mental health services in these territories.

## 4. Discussion

Mental health service provision in rural areas has been analyzed previously in Australia (38) and in other international remote areas (7), but, to our best knowledge, this is the first international comparison of mental health provision in rural areas. The main contextual differences between the study areas were the presence of medium-size main population centers in the two Western European areas, and the greatest surface of the two Australian areas. Our findings highlight clear differences between the service provision of four rural areas in Australia and Western Europe. A higher number of types of care and greater diversity due to both differential characteristics was expected to favor the European areas (39, 40). The city of Trondheim raised the care availability and diversity in the area of Norway, but, in contrast, the city of Lleida did not have the same effect on the Lleida health region in Spain.

The similarities and differences in the care patterns found in this study at two levels, between areas and international regions, underscore the relevance of using standardized methods and tools to gather local evidence to inform service planning.

The main difference in the rural service provision between both world areas was the availability of day care. Previous studies had already showed the scarce availability of this type of care in Australia, both for rural and urban areas (38, 41). It does not necessarily indicate a poorer service pattern in comparison to Europe since day care is compensated by stronger availability of social outpatient care, but the implication of this finding deserves further analysis. The lesser provision of day care services in Australian areas could be related to the context. Their bigger areas and poorer accessibility mean that the population threshold for this type of services is not reached. Moreover, it could also explain the higher availability of mobile outpatient services in South West, but not in Central Tablelands. The lower level of day care in Australia may be a substantive difference with Europe as this gap was also identified in urban areas (41).

The National Mental Health Commission described the Australian service model as complex and fragmented (42). These characteristics are more accentuated in rural environments due to higher number of smaller providers (8). However, our findings only showed a higher weight of care coordination (included in the Accessibility to care DESDE-LTC branch) in one case (South West region), even though it was remarkably higher than the other area with availability of this type of service (Lleida).

On the contrary, the residential care pattern did not show such differences across the two international regions. Lleida, as other Catalonian areas, has a community mental health system with lower availability of beds in core healthcare services such as hospitals (21, 43), while the residential pattern in Sør-Trøndelag is just the opposite, with higher availability of hospital beds both in the main hospital and in the local hospitals. This study has found a similar heterogeneity between the Australian areas, where Central Tablelands has more available hospital beds per inhabitant than in South West, where community residential bed availability is slightly higher.

Alternatives to hospitalization, a key pattern of community mental healthcare (19), were uncommon in the European areas and absent in the Australian cases. This is also the case in urban areas in Australia, although in Europe access to such alternatives was also uncommon (37). In both international regions acute hospital care may be filling gaps that could be covered more efficiently by less intensive more community-based services (44). A similar study in Chile indicated that the rural area of Maule also showed this lack of alternatives to hospitalization (45). Also higher rates of supported accommodation and day centers were found in the rural areas in comparison with the urban ones in Australia (38). However, community residential care was less available in European rural areas than in the urban ones (36, 37, 43).

The balance of health/social care and the diversity of care had no clear pattern that could differentiate between the selected areas in the two world regions. The role of non-health services in European catchment areas was described as heterogeneous in previous studies (21, 37). Our findings point out a similar conclusion for Australia.

This piece of research is part of the GLOCAL project (27) and its healthcare ecosystem approach to provide comparable information on service patterns of a number of health districts from different mental health systems around the world. Indeed, both the readiness analysis and the impact analysis show the usefulness of the healthcare ecosystem approach, including the DESDE-LTC tool and the related local atlases and directories, for service planning, and encourages to extend it to other geographical areas and service sectors (e.g., justice or education services). GLOCAL datasets enable researchers to carry out international comparisons providing informed evidence to support managers, planners, and decision-makers in mental health planning and policy. This is particularly relevant in Australia where rural service data sets have been described as incomplete, disjointed and limited (8).

These findings provide evidence on the usefulness of this method and tools to map mental health care in rural areas, but it does not indicate that some systems are better than others. Care systems are adaptative to time and place context, and one-size-fits-all service models would be inappropriate, especially in rural areas. Furthermore, comprehensive and comparable mental health service data can be the source to carried out data analytics and models to assess the performance of mental health ecosystem and the potential impact of a specific policy, and identifying benchmark and target-for-improvement catchment areas (12, 46).

This study has several limitations. A demonstration study of the usability of DESDE-LTC to estimate the workforce capacity has been published elsewhere (47). As expected, the study areas, even though predominantly rural, have very different characteristics. The Australian areas do not have medium-size cities as main population centers unlike the two European areas. Although the difficulty of defining homogenous rural areas should be taken into account, this does not preclude the utility of DESDE-LTC for comparative analysis. Moreover, it would be helpful to include rural areas from other international regions to give a more complete picture of the rural service provision around the world. The present study could be replicated in other international regions since the DESDE-LTC system has been successfully used in studies from all the continents (28), proving its adaptability and validity in other contexts. This study uses comparable service data in rural contexts. Data were also collected in different years within a time span of 5 years (2012–2016). This limitation indicates the importance of carrying out large scale analysis of the service provision at a single point of time as well as follow-up analyses of the mental health system over time to document changes. This is not possible without a long-term commitment of health agencies in the evaluation and monitoring of service provision, whenever possible given the short-term political horizons.

## 5. Conclusion

This research has demonstrated the feasibility of international comparison of the mental health adult service provision in rural areas in highly divergent national and world regions. The findings revealed differences, but also similarities in the comparison of the care pattern between the study areas in these regions. Day care had a greater weight in the rural European pattern of care, while it was replaced by social outpatient care in Australian areas. In contrast, care coordination was more common in Australia which could indicate a more fragmented system that needs separate navigation services. The balance of hospital and community residential care had no difference between the two world regions, or across catchment areas. However, rural areas in both world regions showed common patterns in the scarcity of alternative services to hospitalization. The analysis of the balance of care between health-related and social care and diversity of care or MTC did not indicate differences between rural areas in Europe and Australia. The healthcare ecosystem approach developed in the GLOCAL project (27) was supported by the positive readiness and impact analysis. This study provides information on context and service provision that may inform, directly or through subsequent analyses, the management and planning of these study areas and help to overcome rural healthcare problems identified in the Orange Declaration (8). Future research will require extending the analysis of rural areas at state or country level, using this approach to analyze workforce capacity in rural areas (47), and conducting benchmark and efficiency analysis to improve care provision. This will require a long-term collaboration and engagement with public agencies, as it has been shown in urban and semi-urban care planning (48).

## Data availability statement

The datasets presented in this study can be found in online repositories. The names of the repository/repositories and accession number(s) can be found below: GLOCAL (Global and Local Observation and mapping of CAre Levels): https://www.canberra.edu.au/research/institutes/health-research-institute/glocal.

## Author contributions

LS-C, DP, AR, and DR designed the study. MF, HT-J, and JK collected the data. MG-C, LS-C, and MF classified the service with DESDE-LTC instrument. CG-A and JS-P carried out the statistical analysis and visualization. LS-C and MF evaluated the readiness. CG-A and LS-C evaluated the impact. JS-P wrote the working draft. All authors reviewed and accepted the manuscript.

## References

[1] Russell D Humphreys J Ward B Chisholm M Buykx P McGrail M Helping policy-makers address rural health access problems. *Aust J Rural Health.* (2013) 21:61–71. 10.1111/ajr.12023 23586567

[2] Wakerman J Humphreys J Russell D Guthridge S Bourke L Dunbar T Remote health workforce turnover and retention: what are the policy and practice priorities? *Hum Resour Health.* (2019) 17:99. 10.1186/s12960-019-0432-y 31842946 PMC6915930

[3] Productivity Commission. *Mental Health Productivity Commission Draft Report.* Canberra: Productivity Commission (2019). p. 1–602.

[4] Vardoulakis S Matthews V Bailie R Hu W Salvador-Carulla L Barratt A Building resilience to Australian flood disasters in the face of climate change. *Med J Aust.* (2022) 217:342–5. 10.5694/mja2.51595 35717626 PMC9795877

[5] Fitzpatrick S Perkins D Luland T Brown D Corvan E. The effect of context in rural mental health care: understanding integrated services in a small town. *Health Place.* (2017) 45:70–6. 10.1016/J.HEALTHPLACE.2017.03.004 28288445

[6] Kelly B Lewin T Stain H Coleman C Fitzgerald M Perkins D Determinants of mental health and well-being within rural and remote communities. *Soc Psychiatry Psychiatr Epidemiol.* (2011) 46:1331–42. 10.1007/s00127-010-0305-0 21046069

[7] Salinas-Perez J Gutierrez-Colosia M Furst M Suontausta P Bertrand J Almeda N Patterns of mental health care in remote areas: Kimberley (Australia), Nunavik (Canada), and Lapland (Finland). *Can J Psychiatry.* (2020) 65:721–30. 10.1177/0706743720944312 32720514 PMC7502882

[8] Perkins D Farmer J Salvador-Carulla L Dalton H Luscombe G Salvador-Carulla L The orange declaration on rural and remote mental health. *Aust J Rural Health.* (2019) 27:374–9. 10.1111/ajr.12560 31515882

[9] Judd F Humphreys J. Mental health issues for rural and remote Australia. *Aust J Rural Health.* (2008) 9:254–8. 10.1111/j.1440-1584.2001.tb00431.x 11736851

[10] Hirsch J Cukrowicz K. Suicide in rural areas: an updated review of the literature. *J Rural Ment Health.* (2014) 38:65–78. 10.1037/rmh0000018

[11] Cheung Y Spittal M Yip P Pirkis J. Spatial analysis of suicide mortality in Australia: investigation of metropolitan-rural-remote differentials of suicide risk across states/territories. *Soc Sci Med.* (2012) 75:1460–8. 10.1016/j.socscimed.2012.04.008 22771036

[12] García-Alonso C Almeda N Salinas-Pérez J Gutiérrez-Colosía M Iruin-Sanz Á Salvador-Carulla L. Use of a decision support system for benchmarking analysis and organizational improvement of regional mental health care: efficiency, stability and entropy assessment of the mental health ecosystem of Gipuzkoa (Basque Country, Spain). *PLoS One.* (2022) 17:e0265669. 10.1371/journal.pone.0265669 35316302 PMC8939819

[13] Fernandez A Black J Jones M Wilson L Salvador-Carulla L Astell-Burt T Flooding and mental health: a systematic mapping review. *PLoS One.* (2015) 10:e0119929. 10.1371/journal.pone.0119929 25860572 PMC4393088

[14] Salvador-Carulla L Amaddeo F Gutiérrez-Colosía M Salazzari D Gonzalez-Caballero J Montagni I Developing a tool for mapping adult mental health care provision in Europe: the REMAST research protocol and its contribution to better integrated care. *Int J Integr Care.* (2015) 15:e042. 10.5334/ijic.2417 27118959 PMC4843179

[15] Castelpietra G Simon J Gutiérrez-Colosía M Rosenberg S Salvador-Carulla L. Disambiguation of psychotherapy: a search for meaning. *Br J Psychiatry.* (2021) 219:532–7. 10.1192/bjp.2020.196 33143767

[16] Lukersmith S Taylor J Salvador-Carulla L. Vagueness and ambiguity in communication of case management: a content analysis in the Australian national disability insurance scheme. *Int J Integr Care.* (2021) 21:17. 10.5334/ijic.5590 33776606 PMC7977023

[17] Mayer S Berger M Konnopka A Brodszky V Evers S Hakkaart-Van Roijen L In search for comparability: the PECUNIA reference unit costs for health and social care services in Europe. *Int J Environ Res Public Health.* (2022) 19:3500. 10.3390/IJERPH19063500 35329189 PMC8948969

[18] Furst M Bagheri N Salvador-Carulla L. An ecosystems approach to mental health services research. *BJPsych Int.* (2021) 18:23–5. 10.1192/bji.2020.24 34287396 PMC8274404

[19] Rosen A Gill N Salvador-Carulla L. The future of community psychiatry and community mental health services. *Curr Opin Psychiatry.* (2020) 33:375–90. 10.1097/YCO.0000000000000620 32452944

[20] Sadeniemi M Almeda N Salinas-Pérez J Gutiérrez-Colosía M García-Alonso C Ala-Nikkola T A comparison of mental health care systems in Northern and Southern Europe: a service mapping study. *Int J Environ Res Public Health.* (2018) 15:1133. 10.3390/IJERPH15061133 29857556 PMC6024953

[21] Cetrano G Salvador-Carulla L Tedeschi F Rabbi L Gutiérrez-Colosía M Gonzalez-Caballero J The balance of adult mental health care: provision of core health versus other types of care in eight European countries. *Epidemiol Psychiatr Sci.* (2020) 29:1–10. 10.1017/S2045796018000574 30328401 PMC8061296

[22] Salvador-Carulla L Costa-Font J Cabases J McDaid D Alonso J. Evaluating mental health care and policy in Spain. *J Ment Health Policy Econ.* (2010) 13:73–86. 20919594

[23] Dijkstra L Brandmüller T Kemper T Asfandiyar K Veneri P. *Applying the Degree of Urbanisation. A Methodological Manual to Define Cities, Towns and Rural Areas for International Comparisons.* Luxembourg: European Union (2021). 10.2785/706535

[24] European Commission. *Global Human Settlement - Visualisation.* (2022). Available online at: https://ghsl.jrc.ec.europa.eu/visualisation.php# (accessed June 14, 2022).

[25] Eckert K Taylor A Wilkinson D Tucker G. How does mental health status relate to accessibility and remoteness? *Med J Aust.* (2004) 181:540–3. 15540965 10.5694/j.1326-5377.2004.tb06442.x

[26] Taylor A Carson D Ensign P Huskey L Rasmussen R Saxinger G. *Settlements at the Edge: Remote Human Settlements in Developed Nations.* Cheltenham: Edward Elgar Publishing (2016). p. 1–449. 10.4337/9781784711962

[27] Health Research Institute. *GLOCAL (Global and Local Observation and mapping of CAre Levels).* University of Canberra (2022). Available online at: https://www.canberra.edu.au/research/institutes/health-research-institute/glocal (accessed September 30, 2022).

[28] Romero-López-Alberca C Gutiérrez-Colosía M Salinas-Pérez J Almeda N Furst M Johnson S Standardised description of health and social care: a systematic review of use of the ESMS/DESDE (European service mapping schedule/description and evaluation of services and DirectoriEs). *Eur Psychiatry.* (2019) 61:97–110. 10.1016/j.eurpsy.2019.07.003 31426008

[29] Salvador-Carulla L Alvarez-Galvez J Romero C Gutierrez-Colosia M Weber G McDaid D Evaluation of an integrated system for classification, assessment and comparison of services for long-term care in Europe: the eDESDE-LTC study. *BMC Health Serv Res.* (2013) 13:218. 10.1186/1472-6963-13-218 23768163 PMC3685525

[30] Salvador-Carulla L Poole M Gonzalez-Caballero J Romero C Salinas J Lagares-Franco C Development and usefulness of an instrument for the standard description and comparison of services for disabilities (DESDE). *Acta Psychiatr Scand.* (2006) 114:19–28. 10.1111/j.1600-0447.2006.00916.x 17087812

[31] eDESDE-Ltc Consortium,. *eDESDE-LTC Coding and Classification System.* (2010). Available online at: http://www.edesdeproject.eu/clasification.php (accessed January 13, 2023).

[32] Romero-Lopez-Alberca C Alonso-Trujillo F Almenara-Abellan J Salinas-Perez J Gutiérrez-Colosia M Gonzalez-Caballero J Semiautomated classification system for producing service directories in social and health care (DESDE-AND): maturity assessment study. *J Med Internet Res.* (2021) 23:e24930. 10.2196/24930 33720035 PMC8074989

[33] Alonso-Trujillo F Salinas-Pérez J Gutiérrez-Colosía M González-Caballero J Pinzón Pulido S Jiménez González S Impact assessment of a multisectoral plan for the promotion of health and social wellbeing in Andalusia (Spain). *Gac Sanit.* (2020) 34:615–23. 10.1016/j.gaceta.2019.01.001 30827502

[34] Hopkins J Wood L Bell T Mendoza J Salvador-Carulla L Karklins L *Integrated Atlas of Mental Health and Alcohol and Other Drugs of Western Australia - Volume II Country WA.* Caloundra, Qld: ConNetica and Mental Health Policy Unit (2017).

[35] Hopkins J Salvador-Carulla L Stretton A Bell T McLoughlin L Mendoza J *The Integrated Mental Health Atlas of Western NSW.* Sydney: The Menzies Centre for Health Policy, University of Sydney and ConNetica (2017). 10.13140/RG.2.2.24639.00165

[36] Salvador-Carulla L Serrano-Blanco A Garcia-Alonso C Fernandez A Salinas-Perez J Gutiérrez-Colosía M *Integral Map of Mental Health Resources of Catalonia, 2010.* Barcelona (2013). Available online at: http://canalsalut.gencat.cat/web/.content/home_canal_salut/professionals/temes_de_salut/salut587_mental/documents/pdf/memoria_atles_integral_version_castellana.pdf (accessed January 13, 2014).

[37] Gutierrez-Colosia M Salvador-Carulla L Salinas-Perez J Garcia-Alonso C Cid J Salazzari D Standard comparison of local mental health care systems in eight European countries. *Epidemiol Psychiatr Sci.* (2019) 28:210–23. 10.1017/S2045796017000415 28918762 PMC6998926

[38] van Spijker B Salinas-Perez J Mendoza J Bell T Bagheri N Furst M Service availability and capacity in rural mental health in Australia: analysing gaps using an integrated mental health atlas. *Aust N Z J Psychiatry.* (2019) 53:1–13. 10.1177/0004867419857809 31250654

[39] Ala-Nikkola T Pirkola S Kontio R Joffe G Pankakoski M Malin M Size matters — Determinants of modern, community-oriented mental health services. *Int J Environ Res Public Health.* (2014) 11:8456–74. 10.3390/ijerph110808456 25153471 PMC4143871

[40] Ala-Nikkola T Sadeniemi M Kaila M Saarni S Kontio R Pirkola S How size matters: exploring the association between quality of mental health services and catchment area size. *BMC Psychiatry.* (2016) 16:285–9. 10.1186/s12888-016-0992-5 27520368 PMC4983042

[41] Fernandez A Gillespie J Smith-Merry J Feng X Astell-Burt T Maas C Integrated mental health atlas of the Western Sydney Local Health District: gaps and recommendations. *Aust Health Rev.* (2017) 41:38–44. 10.1071/AH15154 27007640

[42] National Mental Health Commission. *The National Review of Mental Health Programmes and Services.* Sydney, NSW: National Mental Health Commission (2014).

[43] Fernandez A Salinas-Perez J Gutierrez-Colosia M Prat-Pubill B Serrano-Blanco A Molina C Use of an integrated Atlas of Mental Health Care for evidence informed policy in Catalonia (Spain). *Epidemiol Psychiatr Sci.* (2015) 24:512–24. 25226091 10.1017/S2045796014000511 PMC8367369

[44] Thornicroft G Tansella M. The balanced care model for global mental health. *Psychol Med.* (2012) 43:1–15. 10.1017/S0033291712001420 22785067

[45] Salinas-Perez J Salvador-Carulla L Saldivia S Grandon P Minoletti A Romero Lopez-Alberca C. Integrated mapping of local mental health systems in Central Chile. *Pan Am J Public Health.* (2018) 42:e144. 10.26633/RPSP.2018.144 31093172 PMC6385966

[46] Almeda N Garcia-Alonso C Gutierrez-Colosia M Salinas-Perez J Iruin-Sanz A Salvador-Carulla L. Modelling the balance of care: impact of an evidence-informed policy on a mental health ecosystem. *PLoS One.* (2022) 17:e0261621. 10.1371/journal.pone.0261621 35015762 PMC8752022

[47] Furst M Salinas-Perez J Gutiérrez-Colosia M Salvador-Carulla L. A new bottom-up method for the standard analysis and comparison of workforce capacity in mental healthcare planning: demonstration study in the Australian Capital Territory. *PLoS One.* (2021) 16:e0255350. 10.1371/JOURNAL.PONE.0255350 34314451 PMC8315559

[48] García-Alonso C Almeda N Salinas-Pérez J Gutiérrez-Colosía M Uriarte-Uriarte J Salvador-Carulla L. A decision support system for assessing management interventions in a mental health ecosystem: the case of Bizkaia (Basque Country, Spain). *PLoS One.* (2019) 14:e0212179. 10.1371/journal.pone.0212179 30763361 PMC6375615

